# Expanding the genetic spectrum of mitochondrial diseases in Tunisia: novel variants revealed by whole-exome sequencing

**DOI:** 10.3389/fgene.2023.1259826

**Published:** 2024-01-12

**Authors:** Ismail Gouiza, Meriem Hechmi, Abir Zioudi, Hamza Dallali, Nadia Kheriji, Majida Charif, Morgane Le Mao, Said Galai, Lilia Kraoua, Ilhem Ben Youssef-Turki, Ichraf Kraoua, Guy Lenaers, Rym Kefi

**Affiliations:** ^1^ University of Angers, MitoLab Team, Unité MitoVasc, UMR CNRS (Unité mixte de recherche Centre national de la recherche scientifique) 6015 INSERM (Institut national de la santé et de la recherche médicale) U1083, SFR ICAT, University of Angers, Angers, France; ^2^ Laboratory of Biomedical Genomics and Oncogenetics, Institut Pasteur de Tunis, Tunis, Tunisia; ^3^ Tunis El Manar University, Tunis, Tunisia; ^4^ Faculty of Medicine of Tunis, Tunis, Tunisia; ^5^ Research Laboratory LR18SP04, Department of Child and Adolescent Neurology, National Institute Mongi Ben Hmida of Neurology, Tunis, Tunisia; ^6^ Genetics and Immuno-Cell Therapy Team, Mohammed First University, Oujda, Morocco; ^7^ Department of Clinical Biology, National Institute Mongi Ben Hmida of Neurology, Tunis, Tunisia; ^8^ Department of Congenital and Hereditary Diseases, Charles Nicolle Hospital, Tunis, Tunisia; ^9^ Department of Neurology, CHU d’Angers, Angers, France

**Keywords:** mitochondrial diseases, Leigh syndrome, fumaric aciduria, whole-exome sequencing, 3D structural modeling, genetic diagnosis, North Africa, Tunisia

## Abstract

**Introduction:** Inherited mitochondrial diseases are the most common group of metabolic disorders caused by a defect in oxidative phosphorylation. They are characterized by a wide clinical and genetic spectrum and can manifest at any age. In this study, we established novel phenotype–genotype correlations between the clinical and molecular features of a cohort of Tunisian patients with mitochondrial diseases.

**Materials and methods:** Whole-exome sequencing was performed on five Tunisian patients with suspected mitochondrial diseases. Then, a combination of filtering and bioinformatics prediction tools was utilized to assess the pathogenicity of genetic variations. Sanger sequencing was subsequently performed to confirm the presence of potential deleterious variants in the patients and verify their segregation within families. Structural modeling was conducted to study the effect of novel variants on the protein structure.

**Results:** We identified two novel homozygous variants in *NDUFAF5* (c.827G>C; p.Arg276Pro) and *FASTKD2* (c.496_497del; p.Leu166GlufsTer2) associated with a severe clinical form of Leigh and Leigh-like syndromes, respectively. Our results further disclosed two variants unreported in North Africa, in *GFM2* (c.569G>A; p.Arg190Gln) and *FOXRED1* (c.1261G>A; p.Val421Met) genes, and we described the first case of fumaric aciduria in a Tunisian patient harboring the c.1358T>C; p.Leu453Pro *FH* variant.

**Conclusion:** Our study expands the mutational and phenotypic spectrum of mitochondrial diseases in Tunisia and highlights the importance of next-generation sequencing to decipher the pathomolecular mechanisms responsible for these disorders in an admixed population.

## 1 Introduction

Inherited mitochondrial diseases (iMDs) represent a group of neurometabolic disorders recognized as the largest class of inborn errors in metabolism, with an estimated incidence of 1 in 4,300 individuals (Lehmann and McFarland, 2018). They include a large variety of pathologies with a mitochondrial oxidative phosphorylation (OXPHOS) defect as a common denominator. The OXPHOS system is composed of five multimeric protein complexes integrated in the inner mitochondrial membrane to generate cellular energy as ATP (Jarovsky et al., 2006).

Considering the ubiquitous expression of mitochondria in all nucleated cells and their crucial role in the production of 95% of cellular energy, iMDs can affect any tissue and any organ. Particularly, vulnerable are those with high energy requirements such as the brain, skeletal muscles, heart, liver, and endocrine glands (Jarovsky et al., 2006; Nsiah-Sefaa and McKenzie, 2016). Although iMDs have traditionally been viewed as syndromic presentations, they may present overlapping symptoms that extend beyond recognized syndromes, as well as manifest as single-system disorders (Macken et al., 2021). iMDs can also occur at any age with varying severity, ranging from lethal forms in the neonatal period to moderate or atypical forms in adults (Klopstock et al., 2021). These pathologies can be caused by alterations in the mitochondrial DNA (mtDNA) and/or of the nuclear genome, mainly in genes that encode respiratory chain subunits and OXPHOS complex assembly factors and proteins involved in mtDNA replication, transcription, translation, and maintenance (Frazier et al., 2019). Therefore, they present all modes of inheritance, including autosomal dominant, recessive, X-linked with both inherited and *de novo* mutations, and maternal inheritance (Alston et al., 2017). However, the term “mitochondrial diseases” has recently been broadened to include another set of pathologies, called secondary mitochondrial dysfunctions, caused by mutations in nuclear genes not related to the OXPHOS assembly and activity. They affect diverse mitochondrial functions including, among others, protein biogenesis, mitochondrial morphology and dynamics, lipid biogenesis, and the Krebs cycle (Ceccatelli Berti et al., 2021; Baker et al., 2022). iMD diagnosis remains a challenge given their clinical, biochemical, and molecular heterogeneities. The identification of causal genes is consequently a key tool in supporting the clinical diagnosis (Muraresku et al., 2018).

Since 10% of human genes are predicted to have a mitochondrial function, 1,136 nuclear and mtDNA genes have been considered candidate genes for iMDs, according to the MitoCarta database (MitoCarta3.0) (Rath et al., 2020; Alston et al., 2021). Yet today, over 350 nuclear genes are implicated in mitochondrial disorders (McCormick et al., 2018b; Alston et al., 2021). This number is permanently growing due to the consistent application of next-generation sequencing (NGS), which allows the discovery of new causative genes and mutations, with more than 20 novel genes implicated in mitochondrial diseases identified each year (McCormick et al., 2018a).

The aim of this study was to use high-throughput next-generation sequencing and efficient bioinformatics tools to identify variants causing iMDs among Tunisian families from this underrepresented population.

## 2 Materials and methods

### 2.1 Patients

Our study was conducted in accordance with the principles outlined in the Declaration of Helsinki and following the Institutional Review Board recommendations. It was approved by the Ethical Committee of the Institut Pasteur de Tunis (Tunisia) (Registration Number IRB00005445; reference 2017/28/ILR16IPT). Five patients of Tunisian origin with clinical features suggestive of a mitochondrial disease were recruited from the Department of Pediatric Neurology, Mongi Ben Hamida National Institute of Neurology, Tunisia. The diagnosis was suspected based on the clinical and radiological presentation suggestive of an iMD, as well as biochemical abnormalities indicating possible mitochondrial dysfunction. As index cases were younger than 18 years of age, written consent was signed by the parents. Blood samples were obtained from all patients and their parents.

#### 2.1.1 Patient 1

**FIGURE 1:**
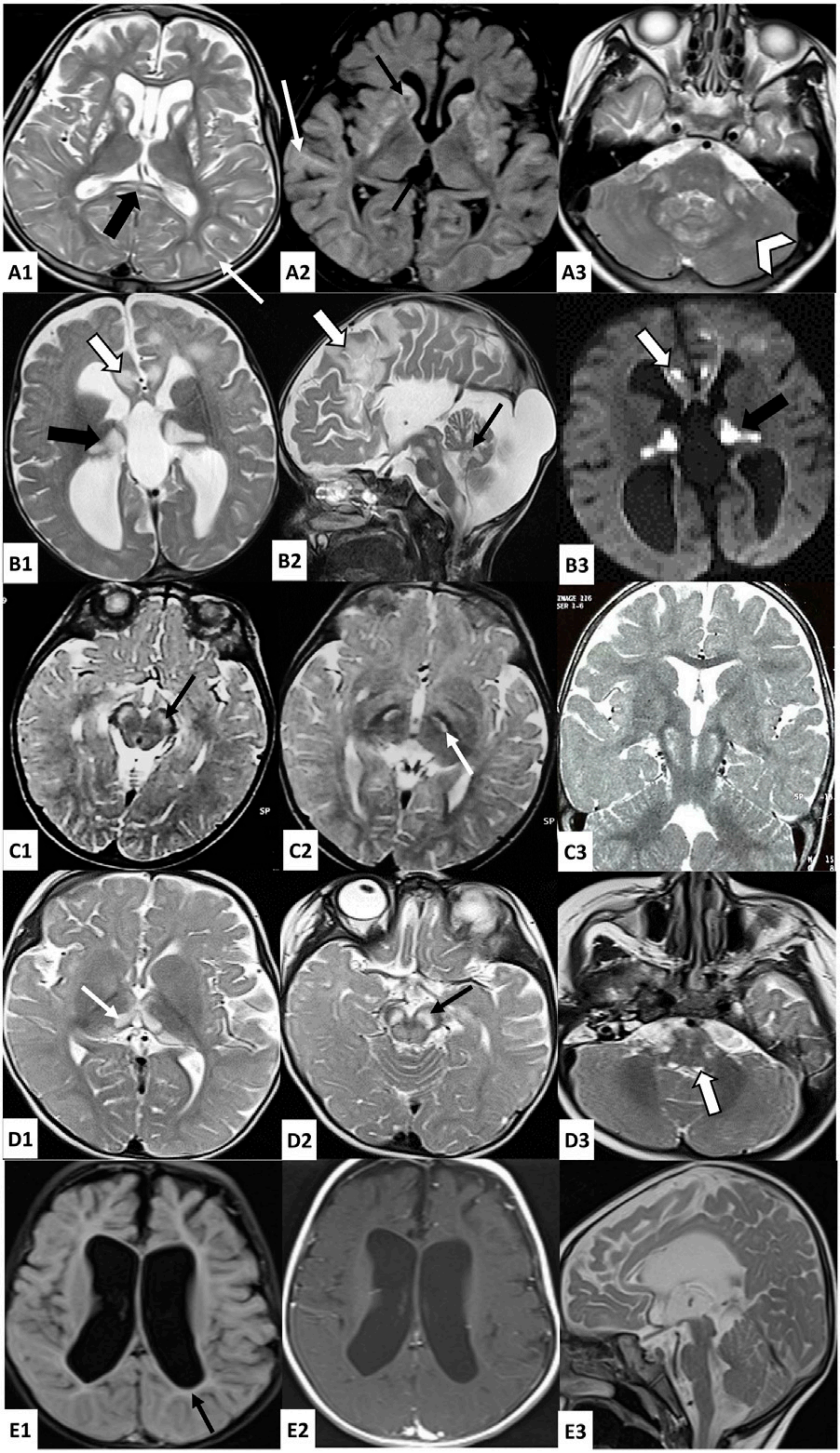
Brain MRI findings in investigated patients. A: Axial T2 **(A1, A3)** and FLAIR **(A2)-**weighted images of patient 1 brain MRI showing bilateral and symmetrical striatal hyperintensities, with the presence of cavitations (thin black arrow), associated with T2 and FLAIR hyperintensities of the periventricular and subcortical white matter (thin white arrow), splenium of the corpus callosum (thick black arrow), middle cerebellar peduncles (thick white arrow), and cerebellar white matter (white arrowhead); B: Axial (B1-B3) and sagittal (B2) brain MRI of patient 2 showing bilateral hyperintense lesions in T2 **(B1, B2)** and diffusion **(B3)-**weighted images affecting the cortex and subcortical white matter in frontal areas (thick white arrow), the thalami (thick black arrow), the midbrain, the pons, the medulla oblongata, and the cerebellum (thin black arrow), as well as cortico-subcortical atrophy predominant at the subtentorial level and agenesis of the corpus callosum; C: Axial **(C1, C2)** and coronal **(C3)** T2-weighted images of patient 3 brain MRI showing bilateral and symmetrical hyperintensities affecting the cerebral peduncles (thin black arrow) and subthalamic nuclei (thin white arrow); D: Axial **(D1–D3)** T2-weighted images of Patient 4 brain MRI showing bilateral and symmetrical hyperintensities affecting the thalami (thin white arrow), the midbrain (thin black arrow) and the posterior part of the medulla oblongata (thick white arrow); E: Axial **(E1, E2)** and sagittal **(E3)** brain MRI of patient 5 showing significant subcortical atrophy with the enlargement of the third and lateral ventricles, mild hyperintensities of the periventricular white matter in FLAIR-weighted image (E1) which are isosignal in T1-weighted image (E2), and hypoplasia of the brainstem.

Patient 1 (P1) is a 13-year-old girl, born to a non-consanguineous couple after a full-term pregnancy and normal delivery. The onset of the disease started when P1 was 1 year old, marked by a delay in psychomotor acquisitions, followed by abnormal eye movements. At the age of 3 years and 8 months, she had two generalized tonic–clonic seizures with ocular revulsion. After 1 month, she was admitted to the neuropediatric department at the National Institute of Neurology (NIN) of Tunis in view of a psychomotor delay. A neurological examination revealed a quadripyramidal syndrome, cerebellar syndrome, horizontal nystagmus, and dystonia. Cerebral magnetic resonance imaging (MRI) showed a necrotizing striatal involvement associated with diffuse cortico-subcortical atrophy and pontocerebellar signal hyperintensities. A doublet lactate peak was observed using spectroscopy (Figure 1). Laboratory examinations revealed lactic acidemia (5.28 mmol/L; normal value 0.5–2.2 mmol/L) and elevated lactate levels in the cerebrospinal fluid (CSF) (3.26 mmol/L; normal values < 2 mmol/L), while plasma amino acid chromatography was normal. Electroencephalography (EEG) showed a slowed background rhythm with no paroxysmal pattern. Eye fundus examination was normal. Muscle biopsy disclosed small mitochondrial overload on cytochrome oxidase/succinate dehydrogenase (COX/SDH) stain with the absence of ragged red fiber. The progression of the disease was marked by the onset of myoclonus and generalized dystonia, which were treated with trihexyphenidyl but without significant improvement. Seizures were initially treated with valproic acid, resulting in the appearance of tremors and drowsiness. As a result, the treatment was switched to carbamazepine, which successfully controlled the epilepsy. Additionally, P1 received carnitine, thiamine, and coenzyme Q10, but these did not significantly improve her condition.

#### 2.1.2 Patient 2

Patient 2 (P2) was a girl born from a second-degree consanguineous marriage after a full-term normal delivery. Since birth, she exhibited a weak cry and had difficulties with feeding. At the age of 5 months, she started vomiting up to seven times a day, accompanied by motor retardation and loss of babbling, for which she was admitted to the pediatric department. A clinical examination revealed hypotonia and psychomotor delay. She underwent cerebral computed tomography, which showed bifrontal, thalamic, and bulbar hypodensities, cerebellar hypoplasia, and agenesis of the corpus callosum. Then, she was referred to the NIN neuropediatrics department at the age of 7 months. She presented no ocular or auditory pursuit, weak cry, axial hypotonia, spastic tetraparesis, axial dystonia, divergent strabismus, horizontal nystagmus, and severe hypotrophy. Laboratory investigations revealed high lactate levels in blood (5.97 mmol/L; normal value 0.5–2.2 mmol/L) and CSF (3.88 mmol/L; normal values < 2 mmol/L). Brain MRI indicated bilateral and symmetrical supratentorial and subtentorial signal abnormalities, with a lactate doublet peak on spectroscopy images of cortico-subcortical, cerebellar, and brainstem atrophy and a complete agenesis of the corpus callosum (Figure 1). Electromyography revealed a slight decrease in motor action potential amplitudes in the median and ulnar nerves. Plasmatic amino acid chromatography and eye fundus were normal. She died at 18 months under unexplained circumstances.

#### 2.1.3 Patient 3

The proband (P3) is a 15-year-old male born to a healthy first-degree consanguineous couple, after a normal pregnancy and delivery. He had two generalized tonic–clonic seizures at the age of 9 months treated with valproic acid. The evolution was marked by psychomotor regression and the persistence of seizures. Since the age of 15 months, episodes of abnormal posture of the fingers and toes lasting 1–2 min were noted, and they disappeared spontaneously. He was admitted to the NIN neuropediatric department at the age of 3 years. A clinical examination revealed axial hypotonia, spastic tetraparesis, and nystagmus. Eye fundus showed optic atrophy, and auditory-evoked potentials were impaired, suggesting hearing loss. He had a high level of lactate in the CSF (4.4 mmol/L; normal values < 2 mmol/L) and an elevated lactate-to-pyruvate ratio of 36.78 in the serum. MRI showed bilateral and symmetrical subthalamic T2 hyperintensities affecting cerebral peduncles and subthalamic nuclei with lactate, as shown by spectroscopy. Plasmatic amino acid chromatography revealed a slight increase in alanine and threonine levels, while chromatography showed that the urinary organic acid level was normal. The evolution was marked by an episode of altered consciousness occurring simultaneously with a pulmonary infection at the age of 26 months. He was admitted to pediatrics, where investigations revealed metabolic acidosis and elevated transaminases, leading to the switch from valproic acid to phenobarbital. The disease course was characterized by a gradual improvement in psychomotor development. At the age of 4 years and 7 months, a muscle biopsy with an ultrastructural study was conducted, revealing the proliferation of mitochondria with abnormal shapes, along with osmiophilic inclusions and mild lipid overload. Subsequently, the patient received treatment with carnitine, coenzyme Q10, and vitamins, along with a prescription for motor and speech therapy.

At the age of 5 years, he showed an improvement in psychomotor abilities by acquiring assisted walking and syllabic language. He returned for consultation at the age of 11 years with a worsened clinical presentation. He lost his ability to walk and to control his head and presented axial hypotonia, strabismus, and spastic tetraparesis. The evolution was marked by the development of scoliosis.

#### 2.1.4 Patient 4

Patient 4 (P4) is a 3-year-old girl born to a first-degree consanguineous couple after a full-term pregnancy and vaginal delivery. The disease started at the age of 5 months, marked by the observation of spontaneous and rapid bilateral conjugate eye movements initially neglected. She also exhibited a bad ocular pursuit at the age of 7 months associated with delayed psychomotor acquisitions. She was referred to the neuropediatric department at the age of 10 months in view of a psychomotor delay. A neurological examination revealed axial hypotonia, generalized dystonia, and nystagmus. A brain MRI examination was performed and revealed bilateral and symmetrical lesions in the substantia nigra, thalami, quadrigeminal tubercles, and posterior bulb, with a lactate peak revealed by spectroscopy (Figure 1). Transesophageal echocardiography showed that the left ventricle was moderately dilated and hyperkinetic. Eye fundus was normal. The biochemical analysis revealed high lactate levels in blood (5.87 mmol/L; normal value 0.5–2.2 mmol/L). She was treated with carnitine, coenzyme Q10, thiamine, and biotin. The evolution of the disease was marked by an improvement in dystonia, along with the persistence of hypotonia, babbling language, and the observation of a ptosis at the age of 15 months.

#### 2.1.5 Patient 5

Patient 5 (P5) is a 4-year-old girl born to first-degree consanguineous healthy parents after full term and a normal delivery. Her family history included the deaths of two siblings, a brother at 8 months and a sister at 1 day under unexplained circumstances, as well as two miscarriages at 8 months and 6 months. In the first months of her life, she presented with a psychomotor delay. She did not acquire either the sitting position or the head control, with the absence of babbling. She also displayed chronic constipation, recurrent vomiting, weak cry, and dysphagia. At 6 months of age, hypotonia and severe psychomotor retardation were observed. One year later, she presented recurrent seizures. A clinical examination revealed no eye pursuit, global hypotonia, abolished reflexes, strabismus, nystagmus, and slight dysmorphic features. Brain MRI revealed cortico-subcortical atrophy, brainstem hypoplasia, and periventricular white matter signal abnormality (Figure 1). Electromyoneurography showed mixed axonal and myelinating neuropathy affecting the four limbs. Abdominal ultrasonography images, auditory-evoked potential, and eye fundus examination were normal. CSF lactate measurement revealed lactatorrachia (2.6 mmol/L; normal values < 2 mmol/L), and the urinary chromatography of organic acids showed an increase in lactic acid levels by 28%. She was prescribed carnitine, coenzyme Q10, and levetiracetam, but poor compliance resulted in only partial control of the epilepsy. At the last clinical evaluation, the patient showed the persistence of global hypotonia and psychomotor delay, poor eye pursuit, and bruxism.

### 2.2 Methods

#### 2.2.1 DNA extraction, quantification, and quality control

Genomic DNA from patients and their parents was extracted from peripheral blood using the FlexiGene DNA Kit (QIAGEN) according to the manufacturer’s instructions. Tthe quality and quantity of the extracted DNA were assessed using the DeNovix DS-11 NanoDrop spectrophotometer and NanoDrop spectrophotometer (Thermo Fisher Scientific).

#### 2.2.2 Whole-exome sequencing and bioinformatics analysis

Whole-exome sequencing (WES) of the five patients was conducted using a BGISEQ-500 sequencer at Beijing Genomics Institute (BGI) Tech Solutions, following the standard protocol (Hong Kong, China). The DNA samples were randomly fragmented into 200–300-bp fragments. After fragment selection, the 3′ ends were repaired, adenosine tails were added, and adapters were attached to both sides of each fragment. Subsequently, PCR amplification was performed to generate DNA libraries. The amplified DNA was captured using the MGIEasy Exome Capture V4 Probe Set (MGI Tech). The captured products were then circularized to produce single-stranded DNA circles, which served as templates for rolling circle amplification to generate nanoballs according to the BGISEQ-500 platform protocol. Raw data quality was checked using FastQC (https://www.bioinformatics.babraham.ac.uk/projects/fastqc/), followed by removing low-quality reads and a trimming step using the SOAPnuke tool (https://github.com/BGI-flexlab/SOAPnuke) (Chen et al., 2017). The remaining reads were aligned to the human genome reference assembly GRCh37/hg19 using the Burrows–Wheeler Aligner tool (BWA V0.7.17) (Li and Durbin, 2009). SNP and INDEL calling were carried out using the GATK HaplotypeCaller tool (v4.1.4.1) (McKenna et al., 2010). The variants were annotated using ANNOVAR (Wang et al., 2010).

Once the files were annotated, a filtering step was conducted to prioritize potential disease-causing variants in nuclear genes that encode mitochondrial proteins and genes associated with mitochondrial functions and operations using VarAFT software (Desvignes et al., 2018). We removed neutral variants and sequencing errors by excluding variants with a depth ≤10 and variants with minor allele frequency (MAF) > 0.01 in gnomAD (https://gnomad.broadinstitute.org/), the 1000 Genomes Project (http://www.1000genomes.org), and the Exome Aggregation Consortium (ExAC) v0.3 (http://exac.broadinstitute.org). We kept only rare functional variants (non-synonymous, non-sense, frameshift, and splice site variants). Then, the non-synonymous variants were filtered after running through 14 prediction tools to retain variants reported as pathogenic by at least 8 of the 14 *in silico* pathogenicity prediction software tools described in a previous study (Dallali et al., 2021). Sequence conservation analysis was performed using the ConSurf server (https://consurf.tau.ac.il/). Splicing variants were evaluated using varSEAK (https://varseak.bio/index.php) and the Human Splicing Finder (www.umd.be/HSF/). We used NMDEscPredictor (https://nmdprediction.shinyapps.io/nmdescpredictor/) to assess frameshift variants and predict the probability of an abnormal transcript being subjected to non-sense-mediated mRNA decay (NMD) or escaping from it (Coban-Akdemir et al., 2018). Each variant was classified according to the American College of Medical Genetics (ACMG) guidelines (Richards et al., 2015). We further evaluated the remaining variants by researching in databases including PubMed (https://www.ncbi.nlm.nih.gov/pubmed), VarSome (https://varsome.com/), LitVar2 (https://www.ncbi.nlm.nih.gov/research/litvar2/), and ClinVar (https://www.ncbi.nlm.nih.gov/clinvar) to check if these variants have been previously reported in the literature and, if so, the associated clinical features.

#### 2.2.3 Sanger sequencing

Sanger sequencing was performed to confirm the presence of the potential causal variations identified by WES and to verify co-segregation among the parents. The oligonucleotide primers were designed using Primer3 software (https://primer3.ut.ee/) followed by amplification by PCR. The PCR products were sequenced on an ABI Prism 3500 DNA automated sequencer (Applied Biosystems, CA, United States) using the BigDye Terminator Cycle Sequencing Reaction Kit v3.1. Sequence analysis was carried out using DNADynamo software (https://www.bluetractorsoftware.com/).

#### 2.2.4 Protein computational analysis

We assessed the impact of the identified variants on the protein 3D structure and function by retrieving the protein structure from the Protein Data Bank (PDB) (https://www.rcsb.org/). In the cases where proteins had not yet been analyzed through nuclear magnetic resonance (NMR) spectroscopy or X-ray crystallography, we utilized the I-TASSER web server to predict their three-dimensional structures. Afterward, the model with the highest confidence score (C-score) was selected. Next, we performed an energy minimization step using YASARA minimization server (Krieger et al., 2009). To further validate the obtained model, quality parameters such as the Ramachandran plot and Z-score were used to assess and evaluate the tertiary structure. Specifically, MolProbity (http://molprobity.biochem.duke.edu/) and ProSA-web (https://prosa.services.came.sbg.ac.at/prosa.php) were used to generate the Ramachandran plot and determine the Z-score, respectively (Wiederstein and Sippl, 2007; Williams et al., 2018). Then, we analyzed the statistics of non-bounded interactions between different atom types using ERRAT (Colovos and Yeates, 1993). The effect of a single amino acid (aa) substitution on the protein stability was performed using I-Mutant 2.0 (https://folding.biofold.org/i-mutant/i-mutant2.0.html) and MUpro (http://mupro.proteomics.ics.uci.edu/). The size, hydrophobicity, and intramolecular interactions involving the wild-type and the mutant residues were analyzed using the HOPE (https://www3.cmbi.umcn.nl/hope/) and DynaMut2 servers (https://biosig.lab.uq.edu.au/dynamut2/). Last, we determined the secondary structure of both wild-type and mutated proteins for newly discovered variants using PSIPred 4.0 (http://bioinf.cs.ucl.ac.uk/psipred).

## 3 Results

**FIGURE 2:**
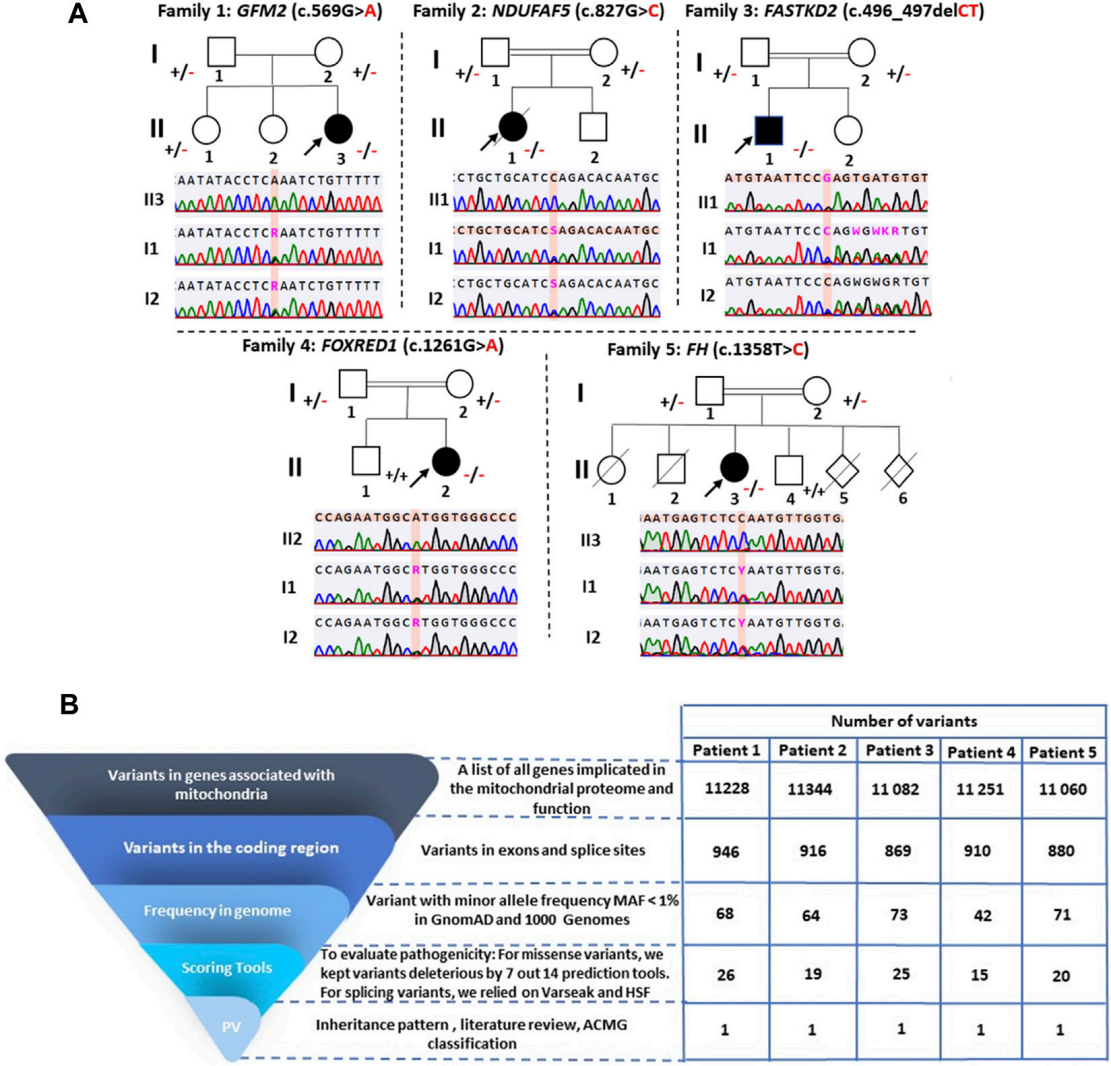
Pedigree analysis of the identified variants and variant filtering strategy used for variant prioritization of exome data. **(A)** Pedigrees of the five investigated families and validation of variants by Sanger sequencing. Squares represent males, circles represent females, double lines indicate consanguineous parents, black-filled symbols and hollow symbols represent affected and unaffected members, respectively, slash indicates a deceased family member, and black arrows indicate the probands. The notation ± indicates heterozygosity for the variant, +/+ represents homozygosity for the normal allele, and −/− signifies homozygosity for the deleterious variant. **(B)** Variant filtering strategy for the WES-generated data. PV: potential variants.

Whole-exome sequencing of the five probands revealed a total of 772,424 variants, with an average of 60,322 homozygous variants and 94,163 heterozygous variants. The mapping rate of the trimmed data ranged from 99.96% to 99.98%. The mean sequencing depth of the target regions was 52.77X, with 97.98% covering >20X. After applying a filtration step involving a gene list implicated in the mitochondrial proteome and function, a total of 55,965 variants remained. Subsequent filtering (Figure 2) identified five homozygous variants in the five patients, consisting of four missense variants and one frameshift deletion (Table1).

**TABLE 1:** Variants identified in patients with mitochondrial diseases and their pathogenicity prediction scores.

		Patient 1	Patient 2	Patient 3	Patient 4	Patient 5
Gender		Female	Female	Male	Female	Female
Gene		*GFM2*	*NDUFAF5*	*FASTKD2*	*FOXRED1*	*FH*
RefSeq		NM_032380.5	NM_024120.5	NM_001136193.2	NM_017547.4	NM_000143.4
cDNA change		c.569G>A	c.827G>C	c.496_497del	c.1261G>A	c.1358T>C
Protein change		p.Arg190Gln	p.Arg276Pro	p.Leu166GlufsTer2	p.Val421Met	p.Leu453Pro
Genotype		Homozygous	Homozygous	Homozygous	Homozygous	Homozygous
dbSNP ID		rs761283105	-	rs1304003703	rs199542988	-
GnomAD exome frequency v2.1		1.6e-5	-	6.572e-6^a^	1.2e-5	-
ACMG classification		Uncertain significance	Uncertain significance	Likely pathogenic	Uncertain significance	Likely pathogenic
Prediction tools/threshold score	SIFT/<0.05	Damaging (0.001)	Damaging (0.001)	Deleterious^b^	Damaging (0.003)	Damaging (0)
	PolyPhen-2 HDIV/>0.453	Deleterious (1)	Deleterious (1)	NA^c^	Deleterious (0.987)	Deleterious (1)
	FATHMM/<0	Tolerated (−0.54)	Deleterious (−2.06)	NA^c^	Deleterious (−2.26)	Deleterious (−6.34)
	MetaLR/>0.5	Deleterious (0.66)	Deleterious (0.79)	NA^c^	Deleterious (0.7551)	Deleterious (0.98)
	MetaSVM/>0	Deleterious (0.5661)	Deleterious (0.7425)	NA^c^	Deleterious (0.6457)	Deleterious (1.042)
	DANN/>0.8	Deleterious (0.9996)	Deleterious (0.9969)	NA^c^	Deleterious (0.9989)	Deleterious (0.9993)
	VEST3/>0.75	Deleterious (0.428)	Deleterious (0.945)	NA^c^	Deleterious (0.222)	Deleterious (0.987)
	CADD/>20	Deleterious (35)	Deleterious (34)	NA^c^	Deleterious (27.9)	Deleterious (32)
	PROVEAN/<-2.5	Deleterious (−3.48)	Deleterious (−6.7)	NA^c^	Neutral (−2.27)	Deleterious (−6.5)
	Mutation assessor/>1.9	Medium (2.84)	Medium (2.895)	NA^c^	Medium (2.595)	Medium (2.255)
	UMD predictor/≥75	Probably polymorphism (57)	Deleterious (99)	NA^c^	Probably polymorphism (63)	Deleterious (84)
	M-CAP/> 0.025	Deleterious (0.0504)	Deleterious (0.1844)	NA^c^	Deleterious (0.2961)	Deleterious (0.8812)
	LRT/D, N, and U	Deleterious	Deleterious	NA^c^	Deleterious	Deleterious
	Mutation taster/A, D, N, and P	Deleterious	Deleterious	Deleterious	Deleterious	Deleterious
Deleteriousness predictions		11/14	14/14	2/2	11/14	14/14

^a^ In gnomAD, genome.

^b^ SIFT, indel.

^c^ NA: not applicable for this frameshift variant.

**FIGURE 3:**
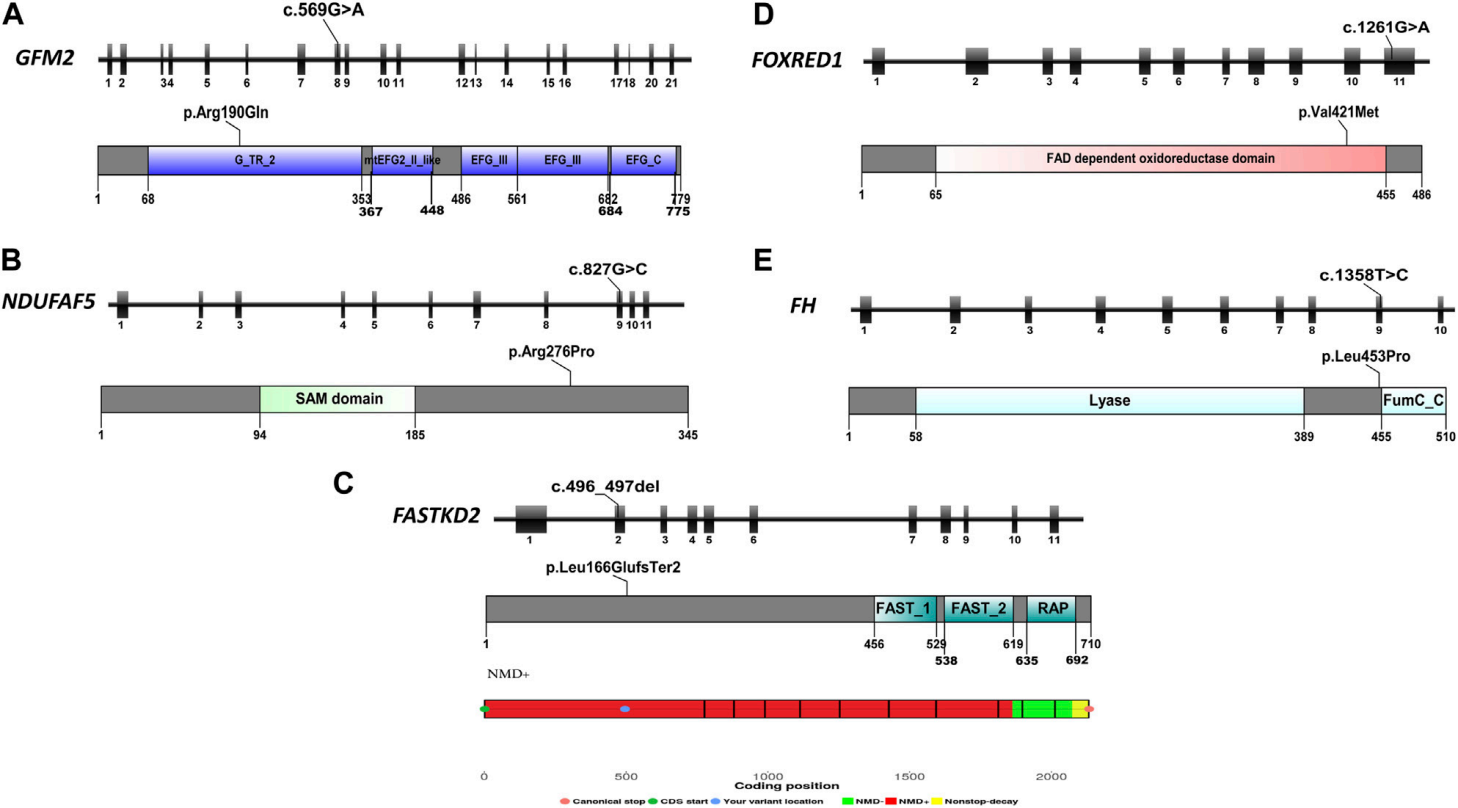
Schematic representation of the five reported genes, displaying their exons, mutation focal points, and respective protein domains. **(A)** Schematic overview of the *GFM2* gene, protein domains, and position of the identified variant in P1; **(B)** schematic overview of the *NDUFAF5* gene, protein domains, and position of the identified variant in P2; **(C)** schematic overview of the *FASTKD2* gene, protein domains, position of the identified variant in P3, and the result of NMD prediction for this variant, indicating its location within the NMD-competent region; **(D)** schematic overview of the *FOXRED1* gene, protein domains, and position of the identified variant in P4; and **(E)** schematic overview of the *FH* gene, protein domains, and position of the identified variant in P5.

**FIGURE 4:**
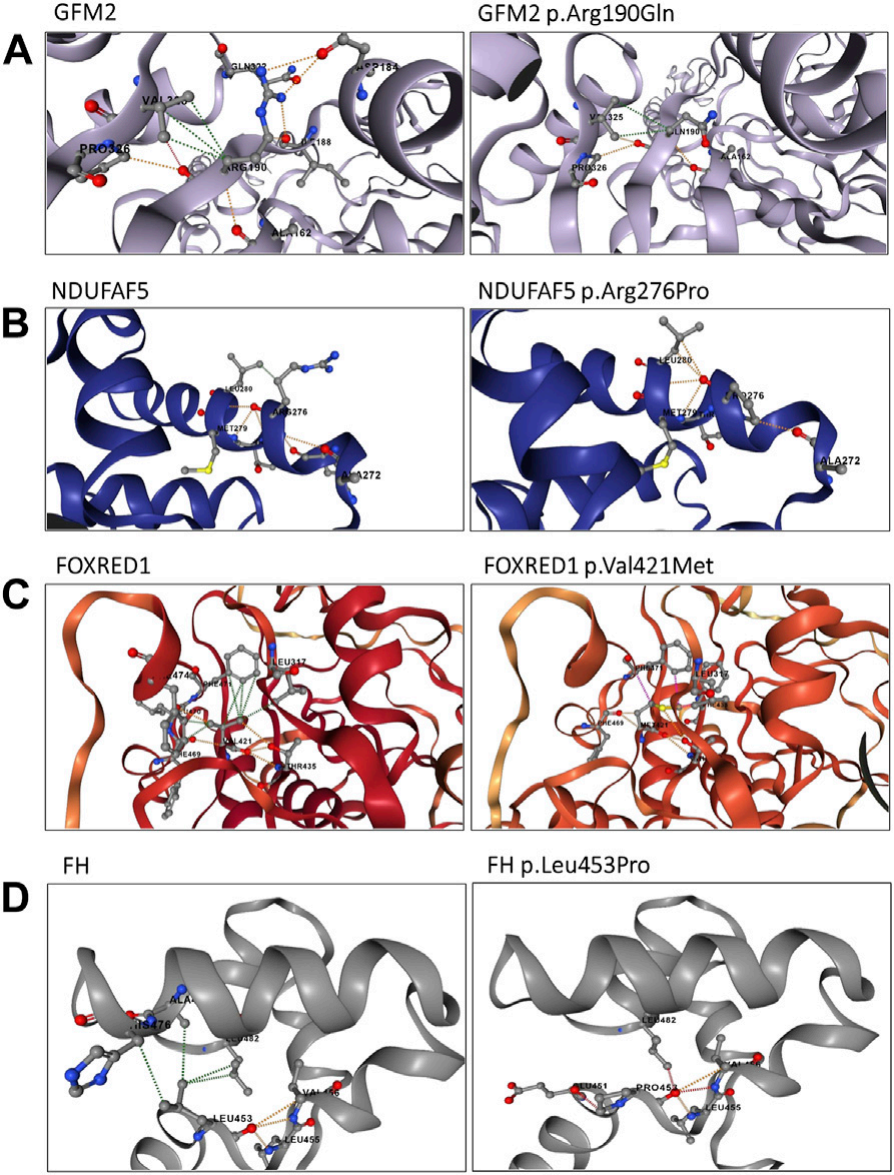
Structural analysis of the identified variants using the DynaMut2 server. **(A)** Structural analysis of the GFM2 p.Arg190Gln variant; **(B)** structure analysis of the NDUFAF5 protein p.Arg276Pro variant; **(C)** intra-atomic interaction analysis of the FOXRED1 protein p.Val421Met variant; and **(D)** intra-atomic interaction analysis of the FH protein p.Leu453Pro variant. Hydrogen bonds are represented by red dashes, polar interactions by orange dashes, and hydrophobic interactions by green dashes.

P1 carried a homozygous variant c.569G>A in the *GFM2* gene (MIM * 606544), leading to the substitution of arginine by glutamine at position 190 (p.Arg190Gln) (Figure 3A). This variant is predicted to be deleterious by 11 of the 14 prediction tools (Table 1). Conservation analysis using ConSurf showed that this variant caused the substitution of a well-conserved residue (=8 according to the conservation scale). I-Mutant 2.0 showed a decrease in protein stability, indicating a negative ∆∆G value of −1.05, and MUpro also indicated a decrease in protein stability with a confidence score of −0.982. The predicted 3D structure of the GFM2 protein passed quality control. Indeed, the Ramachandran plot indicated that 90.6% of residues were in favored regions, with an additional 8.1% in allowed regions. ProSA analysis yielded a Z-score value of −9.75, which falls within the range typically observed for experimentally determined protein structures using X-ray spectroscopy. Furthermore, ERRAT assessment revealed an overall quality factor of 96.089% (see Supplementary File; Supplementary Figure S1A). The mutant residue Gln190 disrupts one hydrogen bond and one hydrophobic interaction with Val325, as well as two hydrophobic interactions with Asp184 and Ile188. It also creates a polar interaction with Val325 (Figure 4A).

The variant has been reported five times in the heterozygous state in gnomAD v2.1.1 (rs761283105), segregating European and Latino/admixed American ethnicities with an allele frequency of 1.6e^−5^. It has never been reported in the homozygous state, and it is classified as a variant of uncertain significance (PM2/PP3/PP5/BP1), according to the ACMG classification. This variant was referenced as a disease-causing variant (CM1718534) by the Human Gene Mutation Database (HGMD). Both parents and her older sister were heterozygous carriers of this variant (Figure 2).

P2 harbored a novel missense variant c.827G>C in the *NDUFAF5* gene (MIM * 612360) leading to the substitution of a charged arginine at position 276 by a smaller, more hydrophobic, and neutral proline (p.Arg276Pro) (Figure 3B). Sanger sequencing revealed that both parents were heterozygous (Figure 2). This variant was predicted to be pathogenic by all the 14 prediction tools (Table 1). Conservation analysis by ConSurf showed that this variant leads to the change of a highly conserved residue (=9 according to the conservation scale), which is predicted to play a functional role (see Supplementary File; Supplementary Figure S2A). MUpro shows a decrease in protein stability with a confidence score of −0.789. I-Mutant 2 confirms this finding, reporting a slightly negative ∆∆G value of −0.03 kcal/mol. Furthermore, three other tools, namely, DUET, SDM, and INPS-3D, indicate an unfavorable ∆∆G of −0.289, −1.41, and −0.856 kcal/mol, respectively. Secondary structure prediction reveals some changes in the protein structure, especially the loss of a β sheet arrangement (Arg42-Phe46) and appearance of α-helix arrangement (Glu254-Asn256) (see Supplementary File; Supplementary Figure S2C). This amino acid alteration could alter the proper function of the NDUFAF5 protein as it also modifies the hydropathic determinants of the protein (see Supplementary File; Supplementary Figure S2B). For the 3D structure, we obtained a model of NDUFAF5 from the I-TASSER server. The Ramachandran analysis indicates that 85.1% of residues are in favorable regions and 11.7% of residues are in allowed regions. The ProSA-web server was used for Z-score validation. It estimates a Z-score of −8.35. This score is within the Z-score range of the experimentally determined protein structure, with a similar size obtained by X-ray spectroscopy and NMR. In addition, the ERRAT results showed an overall quality of 96.4392%. All these results confirm the model’s reliability for conducting stability and mutagenesis analyses (see Supplementary File; Supplementary Figure S1B).

Once the model was properly evaluated and refined, we investigated the effect of the p.Arg276Pro variant on the interatomic interactions. Our analysis showed that this variant disrupted one hydrogen bond with Met279, two polar interactions with Leu274 and Thr278, and a hydrophobic interaction with Leu280. It also creates two new polar interactions with Leu280 (Figure 4). According to the ACMG classification criteria, this variant was classified as uncertain significance (PM2/PP3/BP1). Sanger sequencing revealed that both parents were heterozygous for this variant (Figure 2).

In P3, we identified a homozygous frameshift deletion c.496_497del in the *FASTKD2* gene (MIM *612322), which causes a frameshift and a premature stop codon after two amino acids (p.Leu166GlufsTer2). It is predicted to cause the loss of 542 out of 710 aa (76%), including the highly conserved domains FAST_1, FAST_2, and RNA-binding domain abundant in apicomplexans (RAP) (Figure 3C). This variant is referenced with an allele frequency of 6.572e^−06^ in gnomAD only in the heterozygous state. It was classified as likely pathogenic according to the ACMG classification (PVS1/PM2) and predicted by NMDEscPredictor to cause the loss of normal protein function through non-sense-mediated mRNA decay machinery (Figure 3C).

P4 is homozygous for the variant c.1261G>A in exon 11 of the *FOXRED1* gene (MIM * 613622), causing the substitution of valine by methionine at position 421 (p.Val421Met) (Figure 3). It was confirmed by Sanger sequencing in the patient and his parents. The older brother was confirmed to be a healthy carrier (Figure 2). This missense variant was predicted to be deleterious by 11 tools, and it was classified as a variant of uncertain significance (PM2/PP3/PP5/BP1) according to the ACMG guidelines and as pathogenic by ClinVar (variation ID: 870426) (Table 1). As there is no crystallized FOXRED1 protein available, we proceeded to predict a model and subsequently assessed its quality. The generated Ramachandran plot revealed that out of the 484 amino acids, 416 (86%) were situated in the favored region, while 50 amino acids (10.3%) were within the allowed region. ProSA yielded a score of −8.54, which aligns with the typical Z-score range observed for experimentally determined protein structures obtained through X-ray spectroscopy. Additionally, ERRAT indicated an overall quality rating of 84.31% (see Supplementary File; Supplementary Figure S1C). The substitution of valine by methionine (p.Val421Met) induced a steric clash, which may destabilize the protein. These findings were affirmed by the protein stability prediction tools (Figure 4C). I-Mutant 2.0 displayed a negative ∆∆G value of −2.34, and MUpro showed destabilization of the protein with a confidence score of −0.992. This variant is very rare, exclusively found in the heterozygous state (three times), according to gnomAD (MAF = 1.2e^−5^), and referenced as a disease-causing variant (CM0911501) in the HGMD. This variant segregates in individuals of African, South Asian, and Latino/admixed American ethnicities.

Finally, we found a missense variant c.1358T>C in the *FH* gene (MIM *136850) of P5, causing a leucine-to-proline substitution at position 453 (p.Leu453Pro) (Figure 3). This variant has not been previously described in gnomAD and is referenced as a disease-causing variant (CM117796) in the HGMD. It was attributed a pathogenic effect by 14 prediction tools and classified as likely pathogenic (PP2/PP3/PP5/PM2) according to the ACMG criteria (Table 1). As the protein FH is crystallized with a resolution of 2.10 Å, we retrieved the PDB (PDB accession code: 5D6B) to study the intramolecular interactions of both the wild-type and mutant FH protein. The identified variant resulted in the disruption of four hydrophobic interactions, i.e., two with Leu482, one with His476, and one with Ala475, as well as a polar interaction with Leu482. However, it also led to the creation of three new hydrogen bonds with Glu451, Leu482, and Leu455 (Figure 4D). This variant was confirmed by Sanger sequencing in the proband and in her parents. Her older brother was identified as a heathy carrier (Figure 2).

## 4 Discussion

In Tunisia, diagnosing mitochondrial diseases requires a stepwise approach involving multiple medical disciplines. The preliminary diagnosis is typically established based on the patient’s medical history, physical examination, and laboratory parameters. After completing the clinical evaluation, additional investigations may be performed. The primary approach involves the search for specific lesions using MRI coupled with biochemical analysis to detect energy deficits. Since confirming the disease depends on identifying the causal genetic variation, molecular investigations are conducted through international collaboration. Indeed, until now, the genetic confirmation of mitochondrial diseases in Tunisian patients has relied on international platforms due to limited national resources, notably the absence of a dedicated NGS platform for these disorders. As an alternative, mutation screening for commonly observed mutations in certain mitochondrial diseases is conducted via Sanger sequencing. For instance, the m.3243A>G pathogenic variant in the mitochondrial gene *MT-TL1*, present in approximately 80% of individuals with MELAS syndrome, is screened using this method (Ikeda et al., 2018).

Trio-based WES analysis is the current standard for the molecular analysis of pediatric disorders (Wu et al., 2019). It ensures the identification of *de novo* variants, simplifies prioritization, and provides a higher diagnostic yield. However, we did not use this approach because it is significantly more resource-intensive in terms of cost than focusing solely on the proband’s exome. Our molecular strategy is adapted to the specificity of the Tunisian population. Therefore, we begin by searching for nuclear DNA mutations using WES. If we do not find the causal variant through WES, we proceed to whole-mitochondrial DNA sequencing.

In the present study, we identified five homozygous variants in five Tunisian probands suspected to have mitochondrial diseases. Four out of the five (80%) investigated patients belonged to a consanguineous family. This is not surprising since the Tunisian population has a high rate of consanguinity. Indeed, previous studies have reported a consanguinity rate ranging from 29.8% to 38.0% in Tunisia, which increases the probability of identifying autosomal recessive diseases (Ben Halim et al., 2013; Romdhane et al., 2014; 2019).

P1 carried a homozygous pathogenic variant chr5: 74043556C>T/NM_032380.5: c.569G>A in the *GFM2* gene implicated in combined oxidative phosphorylation deficiency 39 (MIM # 618397). This gene encodes the EF-G2mt protein, which represents a class of guanosine triphosphate hydrolases and is part of the mitochondrial translation complex (Wang et al., 2021). The protein function is to disassemble mitoribosomes in cooperation with the mitochondrial ribosome recycling factor (mtRRF) at the end of translation to ensure continued protein synthesis (Kummer et al., 2021).

The variant c.569G>A was previously described in a British patient in a compound heterozygous state with the variant c.636delA. This patient presented developmental delay, dystonia, and dysarthria, as well as neuroimaging abnormalities in the putamen and caudate nuclei (Glasgow et al., 2017). To evaluate the impact of the variant c.569G>A in the homozygous state, we compared the clinical features of P1 with the previously described case. We observed that P1 exhibited an earlier disease onset at the age of 1 year and presented unreported clinical features, particularly the onset of seizures and nystagmus (see Supplementary File; Supplementary Table S1). Furthermore, our structural analysis revealed that the p.Arg190Gln variant results in the disruption of several intra-atomic interactions (Figure 4A).

To date, four patients harboring variants in the *GFM2* gene, along with the British patient, have been reported in the literature. These cases include two Japanese siblings with Leigh syndrome complicated by multiple congenital arthrogryposis who carried compound heterozygous variants, i.e., c.206 + 4A>G (p.Gly50Glufs4) and c.2029-1G>A (p.Ala677Leufs2) (Fukumura et al., 2015). One patient with microcephaly, simplified gyral pattern, and insulin-dependent diabetes carried the variant c.1728T>A (p.Asp576Glu) (Dixon-Salazar et al., 2012). Finally, a Syrian patient harboring a homozygous variant c.275A>C (p.Tyr92Ser) had clinical features similar to those of the British patient (Glasgow et al., 2017).

All these arguments support the causative effect of this variant in the development of Leigh syndrome observed in P1 and highlighted the impact of this variant in the homozygous state. Further functional studies need to be conducted in order to confirm the pathogenicity of this variant.

Patient 2 carries a homozygous variant chr20: 13797157G>C/NM_024120.5: c.827G>C in the *NDUFAF5* gene that encodes one of the 15 assembly factors of complex I and described to be responsible for mitochondrial complex I deficiency, nuclear type 16 (MIM # 618238). The protein encoded belongs to the family of 7β-strand S-adenosylmethionine-dependent methyltransferases. It processes methyltransferase and arginine hydroxylase activities, ensuring post-translational modifications of its associated proteins (Bi et al., 2021). Indeed, it has been demonstrated that NDUFAF5 catalyzes the hydroxylation of Arg73 within the NDUFS7 subunit of the Q-module and regulates the translation of the ND1 subunit as well as its insertion into the inner mitochondrial membrane (Nouws et al., 2012; Rhein et al., 2016). This functionality has been assigned to its conserved S-adenosylmethionine-dependent methyltransferase domain (SAM domain). The homozygous variant c.827G>C identified in P2 causes the substitution of a highly conserved arginine with a neutral proline, and it has been predicted to be deleterious by 14 bioinformatics tools. Proline is a rigid residue and is known to cause the destabilization of the alpha helix (Kim and Kang, 1999). This is supported by our results obtained using stability prediction tools, as well as the analysis of the hydropathic determinants of the protein (see Supplementary File; Supplementary Figure S2B). Protein modeling showed that p.Arg279Pro induces the loss of one hydrogen bond with Met279, two polar interactions with Leu274 and Thr278, and a hydrophobic interaction with Leu280, which may impair the structure and function of the NDUFAF5 protein (Figure 4B). The identified variant is localized outside the functional SAM domain; however, there is no significant clinical difference between variants located within and outside the SAM domain (Wen et al., 2021).

Reading through the literature, this gene was initially described in 2008 in a consanguineous Egyptian family whose child presented a neonatal form of complex I deficiency and carried the p.Leu229Pro variant (Sugiana et al., 2008). Afterward, several patients have been reported to carry other variants in this gene, whose phenotype was described as Leigh syndrome (Simon et al., 2019; Wen et al., 2021) and isolated bilateral striatal necrosis (Bi et al., 2021). Regarding the disease severity associated with the *NDUFAF5* gene, there is a broad spectrum of variations. These include severe manifestations occurring before birth and resulting in neonatal death, intermediate presentations leading to childhood mortality, and milder forms of the disease that may extend patient survival (Sugiana et al., 2008; Gerards et al., 2010; Saada et al., 2012; Tong et al., 2018). P2 exhibited a severe Leigh syndrome phenotype and died at the age of 1 year and 6 months. It is worth noting that this is the first reported case of a Tunisian patient carrying a variant in this gene. Taking into account these findings, we confirm the diagnosis of Leigh syndrome in P2 and propose the systematic screen of this variant in Maghrebian patients presenting with severe Leigh syndrome. Unfortunately, we could not obtain fibroblasts to conduct functional analysis, but the *in silico* analysis suggested a deleterious effect. Additional functional studies are needed to emphasize the disease pathomechanism related to the discovered *NDUFAF5* variant.

A genetic investigation of P3 revealed the identification of a homozygous variant chr2: 207631913_207631914del/NM_001136193.2: c.496_497del in the *FASTKD2* gene implicated in combined oxidative phosphorylation deficiency 44 (MIM # 618855). This gene encodes a protein belonging to the FASTK family, which includes FASTK and its homologs FASTKD1–5. The FASTKD2 protein is located within the mitochondrial inner membrane and plays a crucial role in ribosome biogenesis, as well as in the processing, maturation, and stabilization of mtRNA (Antonicka and Shoubridge, 2015; Popow et al., 2015). The deletion identified in P3, c.496_497del, causes a frameshift and introduces a premature stop codon at position 167 of the FASTKD2 protein. Consequently, this deletion leads to a loss of the three highly conserved domains: FAST_1, FAST_2, and RAP domains (Jourdain et al., 2015). The functions of these domains are not well explored, but homology studies have demonstrated the involvement of the RAP domain in the RNA-binding protein (Lee and Hong, 2004). Hence, this variant may lead to a complete loss of function as the mRNAs harboring this allele will likely be degraded through the non-sense-mediated decay machinery, as predicted by NMDEscPredictor (Figure 3C) (Alonso, 2005).

To gain further insights into the phenotypic spectrum associated with the *FASTKD2* gene, we compiled information about all reported cases carrying variants in this gene (see Supplementary File; Supplementary Table S2). Summing up the data of the nine patients belonging to seven families with *FASTKD2* variants, we found that there is high variability in both the genetic and phenotypic spectra with lactic acidosis and stroke-like episodes (MELAS) (Yoo et al., 2017; Shah and Balasubramaniam, 2021), infant-onset encephalomyopathy (Ghezzi et al., 2008; Wei et al., 2020), and new-onset refractory status epilepticus (Astner-Rohracher et al., 2023). The disease onset for P3 occurred at 7 months, whereas for all reported patients, it ranged from 6 months to 14 years. Developmental delay and epilepsy were the most frequent clinical features observed in these patients, as well as in P3, followed by optic atrophy, observed in three cases (see Supplementary File; Supplementary Table S2). Notably, P3 presented a Leigh-like syndrome without any episode of stroke-like symptoms. He also exhibited deafness, which has not been reported in previously published cases. Taking into account these data, the c.496_497del variant identified in P3 is responsible for a Leigh-like presentation, thus extending the clinical and genetic spectrum of *FASTKD2-*related iMD.

P4 harbored a homozygous variant chr11: 126147384G>A/NM_017547.4: c.1261G>A in the *FOXRED1* gene. This gene encodes for a 54-kDa mitochondrial complex-I assembly factor (Formosa et al., 2015). Variants in this gene have been linked to mitochondrial complex I deficiency, nuclear type 19 (MIM # 618241). Indeed, the loss of the FOXRED1 function was associated with decreased amounts of fully assembled complex I (Fassone et al., 2010). Previous studies on fibroblast lines from a patient with a deleterious *FOXRED1* variant revealed that this gene is involved in the mid-late stages of complex I assembly (Formosa et al., 2015). According to the literature, the *FOXRED1* variant in P4 was previously reported in an Iraqi patient who showed a decreased level of fully assembled complex I and complex II, along with accumulation of complex I subcomplexes. However, the clinical description of this reported patient has been limited to developmental delay and seizures without a detailed description of the clinical manifestations, as well as MRI and laboratory analysis (Zurita Rendón et al., 2016). The structural analysis of the predicted FOXRED1 protein, in accordance with stability prediction tools, revealed steric clashes that destabilize the protein. P4 presented developmental delay, which represents the most common symptom in *FOXRED1-*related iMD (Hu et al., 2021), and MRI findings were suggestive of Leigh syndrome. Interestingly, P4 did not develop seizures as reported in the Iraqi patient, but he exhibited other features, particularly axial hypotonia, generalized dystonia, nystagmus, and ptosis. Reviewing the literature, 12 deleterious variants have been described in the *FOXRED1* gene since the first variant was reported in 2010 (Fassone et al., 2010). These variants were associated with a diverse range of phenotypes, from infantile encephalopathy (Fassone et al., 2010) to Leigh syndrome (Calvo et al., 2010; Formosa et al., 2015).

To our knowledge, P4 is the second case harboring the variant c.1261G>A and the first case of *FOXRED1* associated with iMD in North African populations.

In P5, we identified a missense variant chr1: 241663769A>G/NM_000143.4: c.1358T>C in the *FH* gene, which encodes fumarate hydratase (EC 4.2.1.2) or fumarase, a key enzyme from the Krebs cycle. It catalyzes the reversible conversion of fumarate to malate through hydration (Zyla and Hodgson, 2021). This enzyme exists in two isoforms: a mitochondrial isoform contributing to the Krebs cycle and a cytoplasmic isoform responsible for the catabolism of fumarate generated by the urea cycle and amino acid catabolism (Peetsold et al., 2021). Mutations in this gene are well documented causing fumaric aciduria (MIM #606812). It is a rare autosomal recessive metabolic disorder characterized mainly by the massive excretion of fumaric acid in urine and a significant decrease in the enzymatic activity of fumarase. To date, over 100 mutations have been reported as associated with fumaric aciduria (Grocott et al., 2020). P5 presented a homozygous variant in the *FH* gene c.1358T>C (p.Leu453Pro), which was previously reported in a Moroccan female with severe fumarase deficiency associated with significant encephalopathy. Her fumarase activity was measured in fibroblasts and was less than 1%, confirming the pathogenicity of this variant (Ottolenghi et al., 2011). She exhibited hypotonia, microcephaly, and psychomotor retardation and died when she was 1 year old. In contrast, P5 is still alive, reaching 4 years of age while sharing some clinical features with the reported patient, including hypotonia and microcephaly. She also presented additional symptoms such as seizures, strabismus, bilateral ptosis, and nystagmus that have not been previously reported in the Moroccan child.

To the best of our knowledge, our study is the first to report a case of fumaric aciduria in Tunisia. As this mutation was also described once in a Moroccan patient (Ottolenghi et al., 2011), it suggests a potential founder effect in North Africa. Therefore, we propose the systematic screening of this variant in suspected cases of fumaric aciduria in North African countries.

Through this study, we emphasize the relevance and significance of early genetic testing in accurately confirming the clinical diagnosis of mitochondrial diseases. This approach ensures improved healthcare management for patients and allows for timely intervention, particularly in the cases where effective treatments are available (Hechmi et al., 2022).

## 5 Conclusion

In this study, we conducted clinical and genetic investigations on five probands with suspected mitochondrial diseases. Our findings revealed two novel variants in the *NDUFAF5* and *FASTKD2* genes, which were associated with the development of Leigh syndrome and Leigh-like syndrome, respectively. Additionally, we identified two previously undescribed variants in *FOXRED1* and *GFM2* genes within North African populations. Finally, we reported the first case of fumaric aciduria in Tunisia. These findings shed light on the clinical and genetic heterogeneity of mitochondrial diseases in Tunisia.

## Data Availability

The original contributions presented in the study are publicly available. These data can be found at: https://www.ncbi.nlm.nih.gov/clinvar/ SCV004031484, SCV004031485, and SCV004171111–SCV004171113.

## Abbreviations

aa, amino acid; ACMG, American College of Medical Genetics; BGI, Beijing Genomics Institute; BWA, Burrows–Wheeler Aligner; COX, cytochrome c oxydase; CSF, cerebrospinal fluid; C-score, confidence score; EEG, electroencephalography; ExAC, Exome Aggregation Consortium; FLAIR, fluid-attenuated inversion recovery; GnomAD, Genome Aggregation Database; HGMD, Human Gene Mutation Database; iMD, inherited mitochondrial disease; I-TASSER, Iterative Threading ASSEmbly Refinement; MAF, minor allele frequency; MELAS, mitochondrial encephalomyopathy, lactic acidosis, stroke-like episodes; MRI, magnetic resonance imaging; mtDNA, mitochondrial DNA; mtRRF, mitochondrial ribosome recycling factor; NGS, next-generation sequencing; NIN, National Institute of Neurology; NMD, non-sense-mediated mRNA decay; OXPHOS, oxidative phosphorylation; PCR, polymerase chain reaction; RAP, RNA-binding domain abundant in apicomplexans; SAM, S-adenosylmethionine dependent methyltransferase; and WES, whole-exome sequencing.
