# Supplementary material for: Expanding the genetic spectrum of mitochondrial diseases in Tunisia: novel variants revealed by whole-exome sequencing
Source: Front Genet. 2024 Jan 12;14:1259826. doi: 10.3389/fgene.2023.1259826 (PMC10811255; doi:10.3389/fgene.2023.1259826)
Supplement: Supplementary file 1 [file DataSheet1.PDF]

Supplementary Material

Expanding the genetic spectrum of Mitochondrial Diseases in Tunisia: novel variants revealed by Whole Exome Sequencing

Ismail Gouiza[1,2,3,4], Meriem Hechmi[2,3], Abir Ziyoudi[3,4,5], Hamza Dallali[2,3], Nadia Kheriji[2,3,4], Majida Charif[6], Morgane Le Mao[1], Said Galai[4,7], Lilia Kraoua[3,8], Ilhem Ben Youssef Turki[3,4,5], Ichraf Kraoua[3,4,5], Guy Lenaers[1,9], Rym Kefi[2,3*]

[1]University of Angers, MitoLab team, UMR CNRS 6015—INSERM U1083, Unité MitoVasc, SFR ICAT, Angers, France

[2]Laboratory of Biomedical Genomics and Oncogenetics, Institut Pasteur de Tunis, Tunis, Tunisia

[3]Tunis El Manar University, Tunis, Tunisia

[4]Faculty of Medicine of Tunis, Tunis, Tunisia

[5]Research Laboratory LR18SP04, Department of Child and Adolescent Neurology, National Institute Mongi Ben Hmida of Neurology, Tunis, Tunisia

[6]Genetics and Immuno-Cell Therapy Team, Mohammed First University, Oujda, Morocco

[7]Department of Clinical Biology, National Institute Mongi Ben Hmida of Neurology, Tunis, Tunisia

[8]Department of Congenital and Hereditary Diseases, Charles Nicolle Hospital, Tunis, Tunisia

[9]Department of Neurology, CHU d'Angers, Angers, France.

*** Corresponding Author:**

rymkefi@pasteur.utm.tn

1. Supplementary Figures

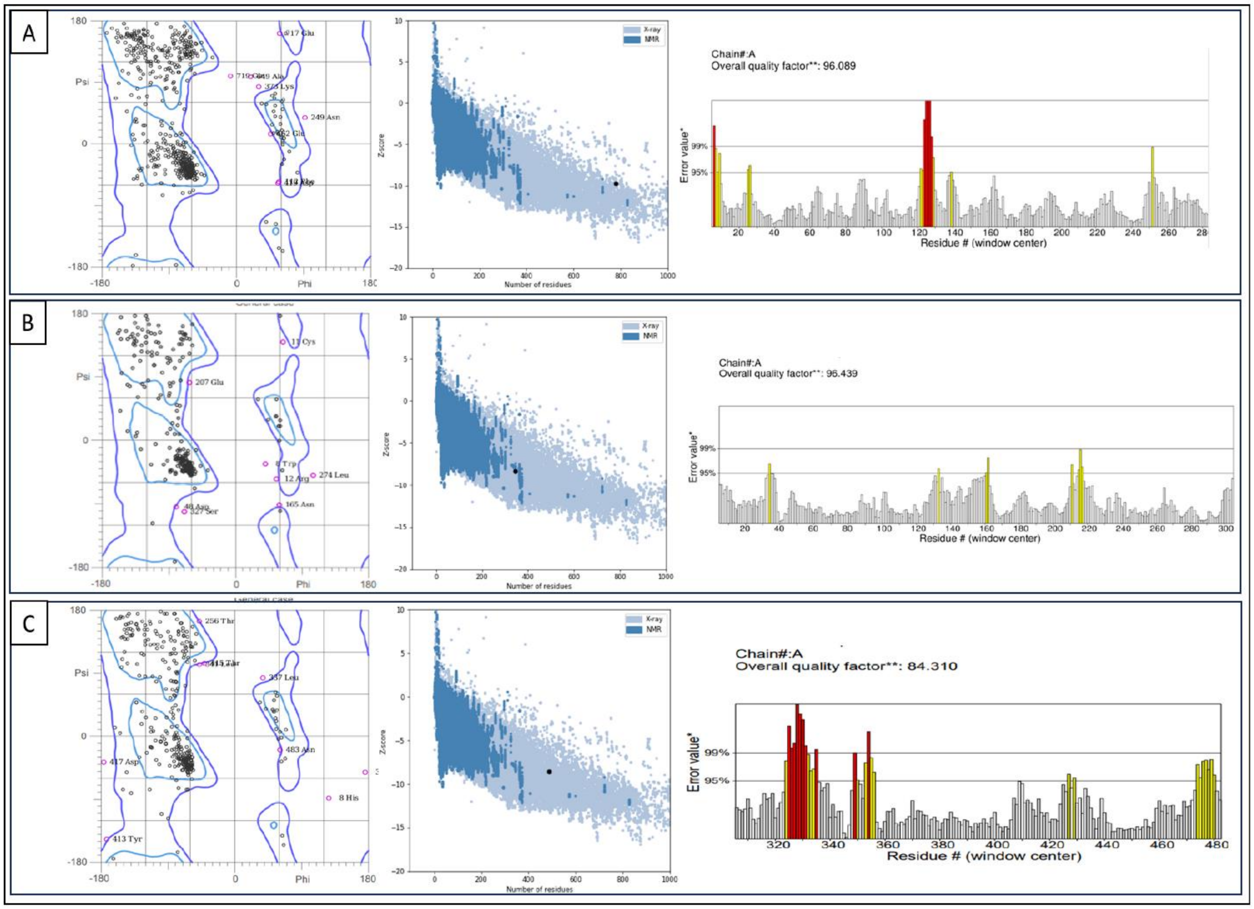

Figure S1: Quality assessment of predicted models

A: ProSA Z-score, Ramachandran plot distribution, and graphical representation of ERRAT output for the GFM2 predicted model. B: ProSA Z-score, Ramachandran plot analysis, and ERRAT quality assessment of the NDUFAF5 predicted model. C: FOXRED1 Z-score prediction model value, Ramachandran plot analysis, and ERRAT output.

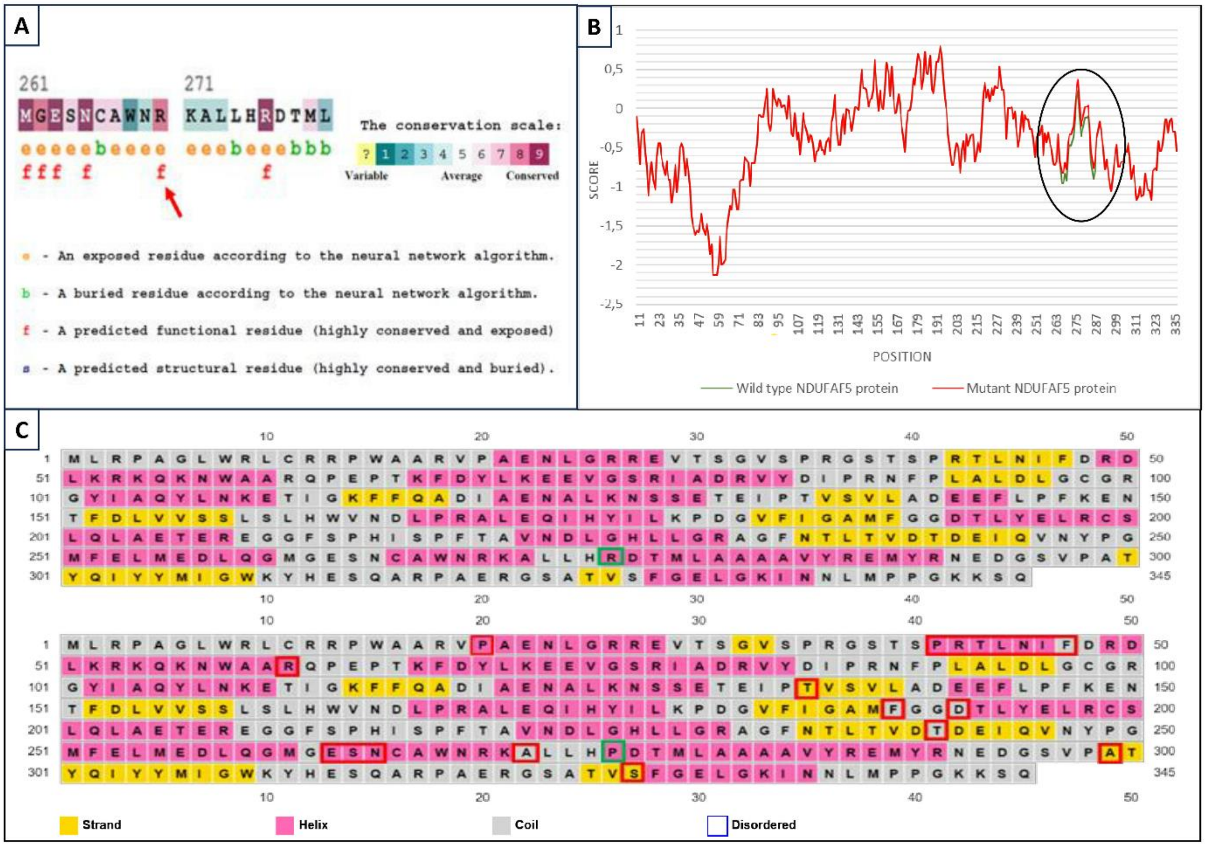

Figure S2: Computational analysis of the novel p. Arg276Pro variant in the NDUFAF5 protein

A: Conservation score of position 276 of the NDUFAF5 protein; B: Superimposed hydropathy plots of the NDUFAF5 amino acid sequence for the normal (green) and mutated (red) proteins using ProtScale output; C: Secondary structure prediction of NDUFAF5 protein: predicted secondary structures for the wildtype and mutant proteins. Red rectangles were used to highlight the sites where differences were observed in the structures of the wildtype compared to the mutant and the green rectangles were used to show p. Arg276Pro variant.

2. Supplementary Tables

Table S1 : Clinical comparison of patients carrying the variant c.569G>A in *GFM2* gene

	**Glasgow et al., 2017**(Glasgow et al., 2017)	**Our study**
**Variant**	c.569G>A, (p.Arg190Gln); c.636delA, (p.Glu213Argfs*3)	c.569G>A, p.(Arg190Gln)
**Age**	11 years	9 years
**Birth history**	Absent	Absent
**Sitting**	NR	12 months
**Walking**	14 months	2.5 years
**Speech**	Dysarthric speech at 5 years	Syllables at 7 years
**Age of onset**	2.5 years	1 year
**Onset clinical symptoms**	Development delay	Development delay
**Nystagmus**	Absent	Present
**Dystonia**	Present	Present
**Seizures**	Absent	Present
**CSF Lactate**	3.4 mmol/L	3,26 mmol/L
**Blood Lactate**	NR	5,28 mmol/L
**Brain MRI**	Bilateral and symmetrical hyperintensities in the caudate, putamen, and cerebellar dentate nucleus. Abnormalities in the corpus callosum and the subcortical white matter of both the cerebral and cerebellar hemispheres with further abnormal areas in the deep white matter	Bilateral and symmetrical bistriatal hyperintensities in T2 and Fluid-attenuated inversion recovery (FLAIR) sequences, accompanied by the presence of cavitations. Hyperintensities observed in the periventricular and subcortical white matter, splenium of the corpus callosum, middle cerebellar peduncles, and cerebellar white matter.

Abbreviations: NR- not reported

frontiers

Table S2: Clinical features of affected individuals with *FASTKD2* variants associated with mitochondrial diseases

	The present study	Astner-Rohrachern et al (2022) (Astner-Rohracher et al., 2023)	Shah et Balasubramaniam (2021) (Shah and Balasubramaniam, 2021)		Wei et al. (2020) (Wei et al., 2020)			Yoo et al. (2017) (Yoo et al., 2017)	Ghezzi et al. (2008) (Ghezzi et al., 2008)	
Age/sex	15 years/male	25 years/male	14 years/female	9 years/male	3 years/female	12 years/female	3.5years/female	33 years/female	16 years/female	NR/male
Origin	Tunisia	Austria	India		China	China	China	Korea	Israel	
Consanguinity	Yes	No	No		No	Yes	No	No	Yes	
Variant	p.Leu166GlufsTer2	p.Arg358Ter	c.991-2A>G (p.?)		p.Leu270fsTer11	p.Arg290Ter	p.Ser621LfsTer14+ p.Arg290Ter	p.Arg176Ter + p.Leu225Pro	p.Arg416Ter	
Birth history	Absent	Absent	Absent	Absent	Absent	Absent	Absent	Absent	Absent	Absent
Onset clinical symptoms	Seizure	Seizure	Seizure	Seizure	Axial hypotonia and<br><br>Dyskinesia (6 months)	Seizure	Development delay	Seizure	Seizure	Seizure
Seizure onset age	9 months	14 years	2,5 years	3,3 years	NR	22 months	1 year	15 years	7 months	1 year
Seizure types	Generalized tonic-clonic	Focal to bilateral tonic-clonic seizure	focal seizures	focal seizures	NR	Generalized tonic-clonic	Generalized tonic-clonic	Generalized tonic-clonic	Generalized tonic-clonic	Generalized tonic-clonic

Development delay	Present	Absent	Present	Present	Present	Present	Present	Present	Present	Present
Other clinical manifestations	Axial hypotonia, spastic tetraparesis, strabismus, scoliosis	Status epilepticus, mild psychomotor slowing, myopathy, spastic atactic gait	Status epilepticus	Hemiparesis	Axial hypotonia, dyskinesia,	Dyskinesia, unconscious shaking of hands, occasional convulsions, sinus tachycardia and hypertrophic cardiomyopathy, status epilepticus	Nystagmus, hypotonia, slurred speech, Stroke-like episode, status epilepticus	Status epilepticus, Dystonia	Hemiplegia	Status epilepticus, strabismus, Dystonia, Hypotonia
Optic nerve atrophy	Present	NR	NR	NR	NR	NR	Present	Present	Present	Present
auditory evoked potential	Delayed	ND	ND	ND	ND	ND	ND	ND	ND	ND
Plasma lactate level	2,88 mmol/l (normal:0.5 – 2.2 mmol/l)	2.2 mmol/l (normal 1.1–2.2 mmol/l)	NR	NR	3.4 mmol/l (normal < 2.1 mmol/l)	1.9 mmol/l (normal < 2.1 mmol/l)	6.3 mmol/l (normal < 2.1 mmol/l	2.2 mmol/l (normal< 1.6 mmol/l)	2.2-3.2 mmol/l (normal< 1.8 mmol/l)	normal
CSF lactate level	4,04 mmol/l (normal<2 mmol/l)	2.2 mmol/l (normal 1.1–2.2 mmol/l)	NR	NR	NR	NR	NR	NR	NR	3.8 mmol/l (normal< 1,8 mmol/l)
COX activity	ND	normal	ND	ND	ND	ND	ND	normal	Reduced in muscle.	ND

EEG	Abnormal	Abnormal	NR	NR	Abnormal	NR	Abnormal	Abnormal	Abnormal	Abnormal
Brain MRI-imaging	Abnormalities in the pallidum, subthalamic nuclei and fornix	Diffusion restriction and Flair hyperintensity right temporo-occipital	Diffusion restriction in the left temporal lobe, insular cortex, and left lentiform nucleus	Abnormal areas of diffusion restriction in the left high frontal, parietal, posteromedial temporal cortex, left occipital lobe, and posterolateral thalamus	Bilateral symmetrical hyperintensity signals in globus palidus, medulla oblongata, and mesencephalon	Bilateral symmetrical hyperintensity signals in globus palidus	Brain atrophy, bilateral symmetrical hyperintensity signals in lenticular nucleus and caudate nucleus	Right occipital lobe infarction	Severe atrophic changes on the right hemisphere	Abnormalities in the left nucleus caudatus, globus pallidus, and crus cerebri

Abbreviations: NR- not reported, ND- not done, EEG- electroencephalograph, CSF- Cerebrospinal fluid, COX- Cytochrome c oxydase
